# Smell or vision? The use of different sensory modalities in predator discrimination

**DOI:** 10.1007/s00265-017-2371-8

**Published:** 2017-09-08

**Authors:** Stefan Fischer, Evelyne Oberhummer, Filipa Cunha-Saraiva, Nina Gerber, Barbara Taborsky

**Affiliations:** 1 Behavioural Ecology, Institute of Ecology and Evolution, University of Bern, Wohlenstrasse 50a, 3032 Hinterkappelen, Switzerland; 2 Department of Zoology, University of Cambridge, Cambridge, UK; 3 Institute of Integrative Biology, University of Liverpool, L69 7ZB, Liverpool, UK; 4 Konrad-Lorenz Institute of Ethology, Department for Integrative Biology and Evolution, University of Veterinarian Medicine, Vienna, Austria; 5 Department of Evolutionary Biology and Environmental Studies, University of Zurich, Zurich, Switzerland

**Keywords:** Threat-sensitive assessment of predation risk, Antipredator behaviour, Olfaction, Perception, Cichlids, *Neolamprologus pulcher*

## Abstract

**Abstract:**

Theory predicts that animals should adjust their escape responses to the perceived predation risk. The information animals obtain about potential predation risk may differ qualitatively depending on the sensory modality by which a cue is perceived. For instance, olfactory cues may reveal better information about the presence or absence of threats, whereas visual information can reliably transmit the position and potential attack distance of a predator. While this suggests a differential use of information perceived through the two sensory channels, the relative importance of visual vs. olfactory cues when distinguishing between different predation threats is still poorly understood. Therefore, we exposed individuals of the cooperatively breeding cichlid *Neolamprologus pulcher* to a standardized threat stimulus combined with either predator or non-predator cues presented either visually or chemically. We predicted that flight responses towards a threat stimulus are more pronounced if cues of dangerous rather than harmless heterospecifics are presented and that *N. pulcher*, being an aquatic species, relies more on olfaction when discriminating between dangerous and harmless heterospecifics. *N. pulcher* responded faster to the threat stimulus, reached a refuge faster and entered a refuge more likely when predator cues were perceived. Unexpectedly, the sensory modality used to perceive the cues did not affect the escape response or the duration of the recovery phase. This suggests that *N. pulcher* are able to discriminate heterospecific cues with similar acuity when using vision or olfaction. We discuss that this ability may be advantageous in aquatic environments where the visibility conditions strongly vary over time.

**Significance statement:**

The ability to rapidly discriminate between dangerous predators and harmless heterospecifics is crucial for the survival of prey animals. In seasonally fluctuating environment, sensory conditions may change over the year and may make the use of multiple sensory modalities for heterospecific discrimination highly beneficial. Here we compared the efficacy of visual and olfactory senses in the discrimination ability of the cooperatively breeding cichlid *Neolamprologus pulcher*. We presented individual fish with visual or olfactory cues of predators or harmless heterospecifics and recorded their flight response. When exposed to predator cues, individuals responded faster, reached a refuge faster and were more likely to enter the refuge. Unexpectedly, the olfactory and visual senses seemed to be equally efficient in this discrimination task, suggesting that seasonal variation of water conditions experienced by *N. pulcher* may necessitate the use of multiple sensory channels for the same task.

**Electronic supplementary material:**

The online version of this article (10.1007/s00265-017-2371-8) contains supplementary material, which is available to authorized users.

## Introduction

Predators are a major selective force shaping the morphology and behaviour of prey animals (Godin 1997). Efficient predation evasion capabilities are crucial for the survival of prey animals (Lima and Dill 1990; Godin 1997). If prey detects the predator first, predator-prey interactions are thought to be characterised by five main steps: (i) prey encounters a predator, (ii) prey detects a predator, (iii) prey recognises the predator, (iv) predator approaches prey and (v) prey starts to evade or attack the predator (Lima and Dill 1990; Kelley and Magurran 2011). Prey species are selected to develop counterstrategies to interrupt the sequence as early as possible as the risk of predation increases with every step in the sequence (Helfman 1989; Godin 1997). The ‘threat-sensitive assessment of predation risk’ hypothesis (Kats and Dill 1998; Brown et al. 2011; Segers and Taborsky 2011; Nersesian et al. 2012) states that the assessment of the current local level of predation threat is a particularly important component of this process. This includes the ability to quickly discriminate dangerous from harmless situations. In support of this hypothesis, several studies reported that aspects of flight responses such as flight speed, flight path and recovery time after a flight response differed when prey individuals were faced with a high or a low threat situation (for reviews see Lima and Dill 1990; Kats and Dill 1998).

In vertebrates, visual and olfactory cues from heterospecifics are an important source of information for prey species to induce appropriate antipredator responses (e.g. Curio 1975; Kelley and Magurran 2003; Webb et al. 2009; Brown et al. 2011; Smolka et al. 2011). As misdirected antipredator behaviours can be costly (Lima and Dill 1990; Chivers and Smith 1998; Chivers and Mirza 2001; Wirsing et al. 2005), prey species should discriminate between dangerous predators and harmless heterospecifics. Visual and olfactory cues can transmit different information contents, however, about type, position and distance of a heterospecific. Most experimental studies testing the threat-sensitive risk-assessment hypothesis compared either how different visual (e.g. Hemmi 2005; Raderschall et al. 2011; Smolka et al. 2011) or different olfactory information (for a review see Kats and Dill 1998) affects the perception of predation risk and the ensuing predation evasion response. In contrast, the relative importance of different sensory modalities governing predator-prey interactions is still poorly understood (Martin et al. 2010; Hale et al. 2017). It has been proposed that the use of sensory modalities may be determined by the ecology of prey species. For instance, several studies suggest that aquatic species rely more strongly on olfaction than on vision when discriminating between harmless and dangerous heterospecifics (Gerlai 1993; Kiesecker et al. 1996; Mathis and Vincent 2000; Ferrari et al. 2010). On the other hand, aquatic environments are particularly prone to variability of the visual and olfactory conditions due to varying turbidity or changing of water currents. Turbidity reduces the efficacy of visual cues, whereas currents may disrupt chemical information. This may make the conditional use of sensory input for predator recognition and discrimination highly beneficial (e.g. Dalesman and Inchley 2008; O'Connor et al. 2015). As a first step of understanding the conditional use of sensory input in natural environments, we first need to investigate the efficacy of each of the different sensory channels in accomplishing predator discrimination.

The cooperatively breeding cichlid *Neolamprologus pulcher* is a well-suited model organism to study the flexible adjustment of predator evasion behaviour to perceived predation risk. In its natural habitat in Lake Tanganyika, East Africa, *N. pulcher* form permanent social groups composed of one breeder pair and typically between 1 and 25 related and unrelated brood care helpers, which inhabit highly structured territories representing safe havens from predators for all group members (Taborsky and Limberger 1981; Heg et al. 2005b). Group living in *N. pulcher* is mainly driven by high ambient predation risk; single individuals or pairs alone cannot sustain a territory or reproduce successfully (Heg et al. 2004; Brouwer et al. 2005; Heg and Taborsky 2010; Zöttl et al. 2013). When group members leave the vicinity of shelters provided within territories, for instance, when feeding or defending the territory borders, they are at high risk to be predated. Their main predator is a highly specialized, large piscivorous cichlid *Lepidiolamprologus elongatus*, which often lurks around *N. pulcher* territories (Hori et al. 1983; Heg et al. 2005a). As many different cichlid species regularly pass by these territories, an early and precise recognition of *L. elongatus* individuals is of utmost importance for an efficient predator evasion in *N. pulcher*. Moreover, *N. pulcher* is a particularly interesting species to study the relative importance of visual vs. olfactory predator detection, because the visibility conditions in its natural environment vary greatly across seasons (Plisnier et al. 1999; Plisnier 2002) and water currents impacting olfactory conditions vary with local weather changes (BT, pers. obs.).

Here we compared the efficacy of the olfactory and the visual sense of *N. pulcher* in discriminating dangerous predators from harmless heterospecifics. We manipulated either the visual or the olfactory perception of dangerous or harmless heterospecifics and evaluated how *N. pulcher* use these cues to adjust their flight responses towards a standardized threat stimulus. More precisely, we applied a standard method to elicit startle responses (dropping an object, see Arnott and Elwood 2009, 2010; Reddon et al. 2013) and combined this with the exposure of either moving pictures or water-borne chemical cues of a harmless herbivore or a dangerous predator. We predicted that *N. pulcher* can discriminate better between a dangerous and a harmless heterospecific if olfactory cues are provided than if they receive visual cues (Gerlai 1993; Kiesecker et al. 1996; Mathis and Vincent 2000; Ferrari et al. 2010). Furthermore, in line with the threat-sensitive risk-assessment hypothesis, we predicted flight responses to be more pronounced when cues of the more dangerous species were presented.

## Methods

### Experimental animals

All experimental fish were taken from the long-term lab stocks maintained at the Ethologische Station Hasli, University of Bern, Switzerland, which were generated from wild caught cichlids originating from Kasakalawe point near Mpulungu, Zambia (8° 46.8490′ S, 31° 04.8820′ E) in 2006. To generate predator cues, we used *L. elongatus* (see above). To obtain cues of a non-dangerous, similar-sized fish, we used the herbivorous cichlid *Ophthalmotilapia ventralis*, which feeds on plankton and grazes the turf cover of rocks (Hori et al. 1983; Konings 1998). Both species occur in sympatry with *N. pulcher* along the rocky shores of the southern part of Lake Tanganyika (Karino 1998; Ochi and Yanagisawa 1998). *O. ventralis* is a maternal mouthbrooder, which spawns in open sand craters and does not defend spaces apart from these craters, and which does not use rocks or crevices for hiding. Therefore *O. ventralis* does not compete with *N. pulcher* for potential spawning or hiding places.

### Rationale of the experiment

To elicit a flight response in *N. pulcher* individuals, we dropped a marble close to a test fish in an experimental tank (for details see below). This standardized threat stimulus is known to reliably elicit startle responses in fish (see Arnott and Elwood 2009, 2010; Reddon et al. 2013). Test fish instantly flee away from the disturbance created by the marble. We created four different types of heterospecific cues to be presented with the threat stimulus: either visual or olfactory cues of either piscivorous predators or harmless herbivores, respectively. We observed whether the escape response was flexibly adjusted towards the different types of risk.

### Production of stimulus cues

To present visual cues, we showed animated pictures of heterospecifics to the test fish using Microsoft PowerPoint by applying a method established by Fischer et al. (2014). The presented stimulus fish shown at the animations had a standard length (SL; from the tip of the snout to the posterior end of the vertebral column, i.e. excluding the tail fin) of 12 cm. We randomly used 2-D animations of six *L. elongatus* and six *O. ventralis*. The image of a stimulus fish was pasted onto a greenish background to simulate natural water conditions. As size reference, the background also contained pictures of nine stones of various sizes (one stone each of 3.9 × 12.4 cm, 1.3 × 2.7 cm, 1.2 × 3.9 cm, 1.0 × 2.1 cm and 0.7 × 1.5 cm size; and two stones each of 2.0 × 6.1 cm and 0.9 × 1.9 cm size [height × width]) presented 5 cm above the bottom of each presentation (Zbinden et al. 2004; Baldauf et al. 2008). We displayed the PowerPoint presentations using a flat screen monitor (Compaq 1520, with 15″ and 1024 × 768 pixels) connected to a PC. *N. pulcher* are known to be able to recognize animated images of conspecifics and heterospecifics from flat screen monitors, and they can distinguish between animated images of predators and herbivores (Fischer et al. 2014; Hess et al. 2016).

In the animations, a stimulus fish entered always head first from the right side, crossed the screen in 30 s and left it at the left side; during the following animation, it re-entered head first from the left side and left the screen at the right side, from where it re-entered again. The presentation was started immediately before the cameras were installed (see below) and lasted for the whole trial. For more details on the production of animated pictures, see Fischer et al. (2014).

To manipulate olfactory cues, we used water from the holding tanks of predators or herbivores, which is a well-established method to simulate the presence of heterospecifics based on olfactory cues (e.g. Abjornsson et al. 1997; Ferrari et al. 2006; Frommen et al. 2011; Segers and Taborsky 2012; Stratmann and Taborsky 2014). It is noteworthy that all involved species, the predatory and the herbivorous stimulus fish and *N. pulcher* were fed the same type of food namely commercial flake food (5 days a week) and commercial frozen plankton food (1 day a week). For the presentation of olfactory cues, we used stimulus water from different tanks containing similar densities of either only *O. ventralis* or only *L. elongatus* of different sizes and ages.

### General set-up

The experiment was done in six 200-l tanks (100 × 40 × 50 cm) divided in two experimental compartments (50 × 40 × 50 cm) by an opaque PVC divider. As the separation between compartments might have been permeable to water, both experimental compartments of a tank were assigned to the same treatment. All experiments were recorded using a video camera (Sony HandyCam HDR-PJ260 8.9 Mega pixels) and the camera of an Apple iPhone 4 s. To allow for a correct analysis of flight paths of fish from 2D-video recordings, the tanks were only filled to a water level of 20 cm to ensure that the flight paths occur predominantly in the two horizontal dimensions. Each compartment contained two flower-pot halves as potential refuges. However, only one of them could be entered by the fish (‘shelter’), while the other was blocked by a transparent plastic foil and was hence unsuitable for hiding. These two types of shelters were introduced in the experimental set-up to test for the ability of fish to choose an accessible shelter in response to a startle stimulus, which should be part of adequate predator evasion behaviours. Only in a single trial a test fish tried to flee into the closed flowerpot indicating that almost all fish were easily able to distinguish the suitable from the unsuitable refuge. Thus, the data did not allow for any statistical inference related to the hypothesis that suitable shelters are used more often when more dangerous cues are present. Nevertheless, it shows that *N. pulcher* are capable of remembering locations with suitable shelters irrespective of the perceived threat level.

Twenty test fish (10 males and 10 females) in a size range of 4.0–4.9 cm standard length), haphazardly caught from several institute’s breeding stock tanks, were used for the experiment. Each test fish experienced five treatments in randomized order, resulting in a within subject design: (i) a predator smell treatment (PS), in which water containing chemical cues of the piscivorous predator *L. elongatus* was spread in the experimental tank; (ii) a non-predator smell treatment (NPS), in which water containing cues from the herbivore, *O. ventralis*, was spread in the tank; (iii) a predator-picture treatment (PP), in which an animated image of *L. elongatus* was presented on the screen and (iv) a non-predator picture treatment (NPP), in which an animated image of an *O. ventralis* was presented; (v) in a control treatment, test fish were shown the background image with stones only and before this presentation, clean tap water was added to the experimental tanks.

The day before each trial started, test fish were transferred to one of the experimental test compartments. Fish were allowed to habituate overnight to the test environment without any visual or olfactory predator cues present. Moreover, 12 h before the trial started the test fish did not receive any food in order to prevent that fish are satiated and do not leave their shelter during the trial. Each test fish experienced one treatment per day to reduce potential carry-over effects from previous experiences and to standardize habituation times between the treatments.

**Fig. 1:**
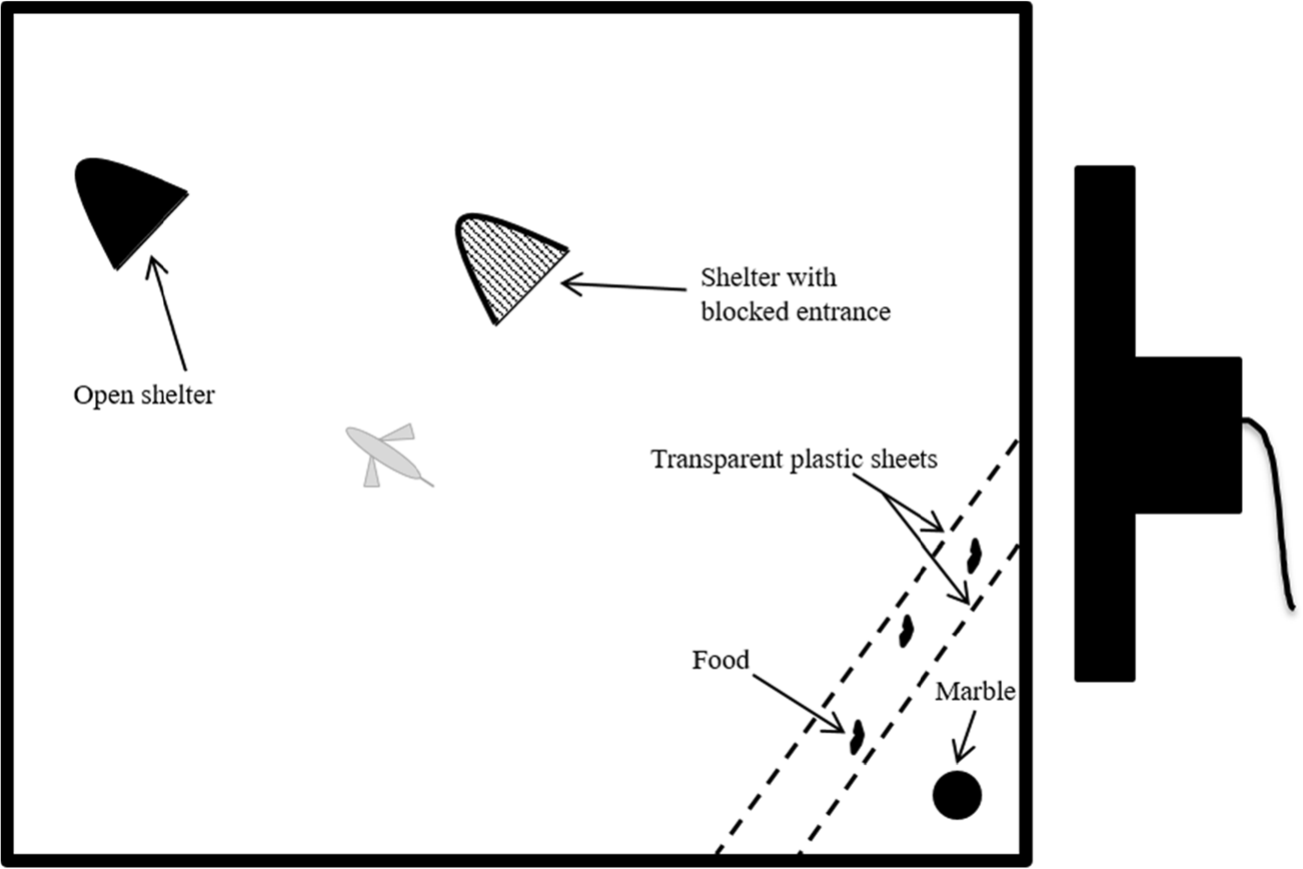
Bird eye view of an experimental compartment. The marble (black circle) dropped always behind the food, which was prevented to float across the experimental compartment by two transparent plastic sheets (dashed lines). Of the two flower-pots halves, one had a blocked entrance. The computer screen (indicated on the left side) displayed the PowerPoint animation (see the ‘Methods’ section)

On the next day we prepared 2.5 L of stimulus water per each 100-L compartment that received an olfactory treatment and 2.5 L clean tap water for each compartment receiving a control treatment. If a trial included an olfaction treatment, the respective treatment water was slowly poured into the compartment from the front side of the tanks using a bucket. If a trial included a visual, a monitor was placed next to the compartment and we immediately started the PowerPoint presentation. This set-up was adopted from an earlier study (Fischer et al. 2014) and assured that test fish could clearly see the PowerPoint presentation. During control trials, we put the monitor in place and poured tap water in the tank. By controlling for visual and olfactory treatments within the same trials, we were able to compare all heterospecific cue treatments to the same baseline in the same statistical analysis, even if with this design fish received also procedures during the control they did not receive during all tests (e.g. the pouring of water did not occur in the visual treatments). After putting visual or olfactory cues in place, we started the cameras to video record the trial and placed a small amount of commercial flake food at the water surface in the front corner of the test compartment that was furthest away from the open shelter (see Fig. 1). This corner was chosen, as it maximised the linear escape distance to the open shelter (approximately 64 cm). To prevent the food from floating across the entire water surface, two transparent plastic strips, fixed slightly below the water surface, confined the flakes in this corner (see Fig. 1). After applying the food, the experimenters (NG or FCS) hid behind a curtain in the aquarium room such that the test fish could not see them. Once a test fish entered this corner, began to feed or was within 5 cm of the food and faced towards it, a coloured glass marble of 2 cm diameter was dropped to elicit the escape response. The latency to start feeding was not different between treatments (*χ* ^2^ (4) = 1.426, *p* = 0.84, *N* = 20), but in general, males approached the food faster than females (*χ* ^2^ (1) = 6.358, *p* = 0.012, *N* = 20). The marble was loosely fitted in a hole drilled in a wooden board that was mounted at a height of 43 cm above the water surface. Below the marble, a removable nail was inserted, which was attached to a string. To drop the marble remotely, the observer quickly pulled the string thereby removing the nail. The marble touched the water surface at a standardized position close to the test fish. The dropping marble created an obvious splash when it hit the water surface and then sunk rapidly to the ground. This disturbance was assumed to simulate an unexpected predator attack (see Reddon et al. 2013). After each trial, a complete water change was done in all experimental tanks.

### Analysis of flight response

The videos were analysed using SimpleMovie X, a freeware video editor program for Mac OS X. We measured four parameters of the flight responses. (i) The latency of each test fish to respond towards the dropping marble. As a reference point for these latencies, we used the time point when the marble hit the water surface, because this moment can be extracted from the videos with high precision. Most test fish responded immediately towards the movement of the marble; actually, all fish did so already before it even hit the water surface, resulting in negative latencies. (ii) We measured the duration of the burst-swim phase as the time between the first response and the end of the flight response (i.e. after the fish stopped at one location or had entered the shelter). (iii) We recorded whether or not test fish entered the shelter after the burst-swim phase. We further analysed the duration of the recovery phase after the escape response, namely (iv) the time test fish spent motionless if the escape response ended outside the shelter or (v) the time test fish spent inside the shelter. Note that the biological interpretation of (iv) and (v) is very similar, but we had to analyse the two variables separately, as they refer to different individuals: some fish ended their flight response inside and some outside the shelter (see below).

To obtain the latencies, burst-swim durations, times in shelter and times spent motionless, we counted the number of frames during which the behaviour occurred. We divided the frame counts by 30 thereby transforming them to seconds as all experiments were recorded with 30 frames per second. NG and FCS analysed the videos while being blind to the treatment.

### Statistical analysis

For statistical analyses, we used R 3.0.2 (R Core Development Team 2013) with the package ‘lme4’ (Bates et al. 2013). To analyse the latencies to respond, burst time and time spent motionless, we used linear mixed models (LMM). As the two components of the response to the marble, latency to respond and burst-swim duration, may not be independent from each other, we corrected for a relationship between the latency and the burst-swim duration; we included the latency as a covariate in the model when analysing the burst-swim duration. To analyse the propensity of test fish to enter the shelter, we used generalized LMM with a logit link function to account for a binomial error structure. The five treatments (C, NPP, NPS, PP and PS) and sex of the test fish were included as fixed effects and the SL of the test fish as a covariate. We conducted four orthogonal comparisons (see Crawley 2007). We set the contrasts of the model to compare first the control treatment against the mean of all treatments in which any stimulus fish cue was present [C vs. (NPP, NPS, PP, PS)]. Second, we compared how test fish responded to predator cues against non-predator cues [(NPP, NPS) vs. (PP, PS)]. Third, we compared the behavioural differences of test fish exposed to an image of a predator or the smell of a predator [PP vs. PS]. Fourth, we compared test fish exposed to the image of a non-predator or the smell of a non-predator species [NPP vs. NPS] (mean values of treatments presented in round brackets were used in the comparisons). To minimize type I errors in our statistical analysis, we used planned contrasts which are defined a priori by the research question and the experimental design (see Abdi and Williams 2010).

Residuals and *Q*/*Q*-plots of all LMM models were visually inspected, and the distributions of residuals were compared to a normal distribution using Kolmogorov-Smirnov and Shapiro tests. If residuals were non-normally distributed, log or square root transformations were applied. To obtain *p* values of model fixed effects, we used the package ‘lmerTest’ (Kuznetsova et al. 2013) or a likelihood-ratio test using the ‘ANOVA’ function in the package car (Fox and Weisberg 2011).

## Results

**Fig. 2:**
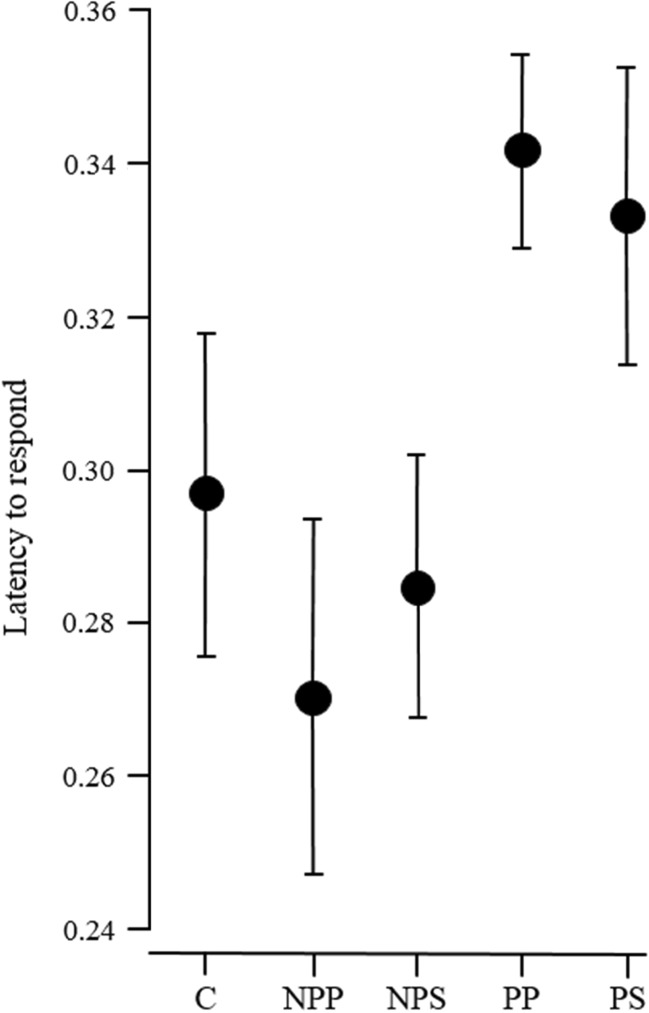
Latency to respond towards the dropping marble (mean ± SE) of test fish in the five different treatments. Note that latencies take negative values as they were measured from the time point when the marble was hitting the water surface, but the fish responded already before the hit. Thus, a negative number of, e.g. − 0.36 along the *y*-axis indicates an earlier response towards the dropping marble than, e.g. − 0.24. For analysis, data were transformed to positive values square-root transformed to achieve normally distributed residuals (see Table 1), and for graphic presentation, they were back transformed to negative values. C = control, NPP = herbivore picture, NPS = herbivore smell, PP = predator picture, PS = predator smell

**Fig. 3:**
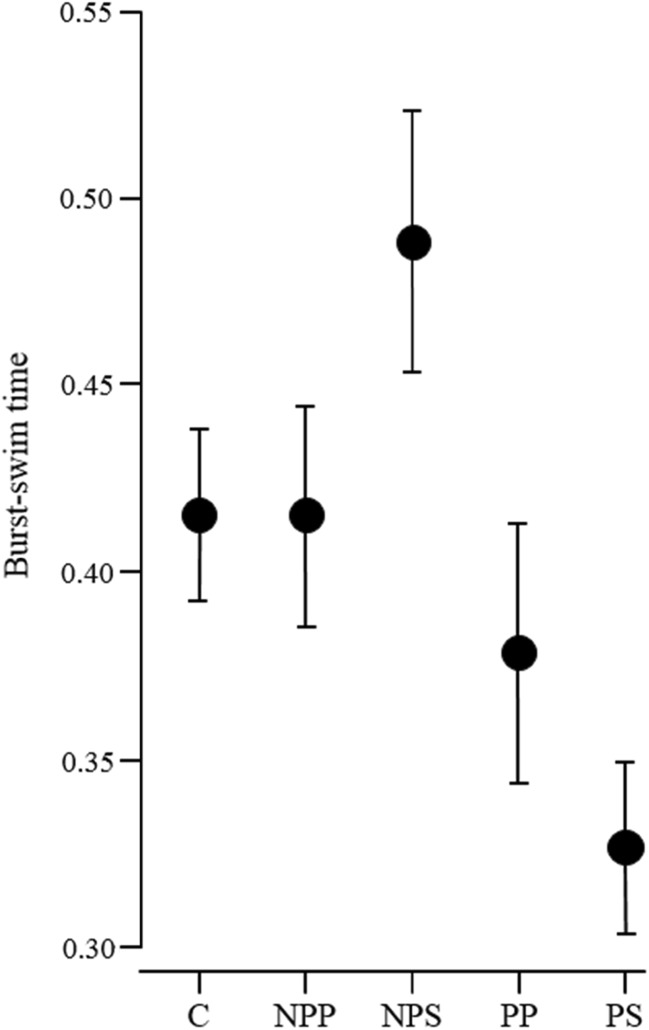
Burst-swim time (mean ± SE) of test fish in the five different treatments. C = control, NPP = herbivore picture, NPS = herbivore smell, PP = predator picture, PS = predator smell

**Fig. 4:**
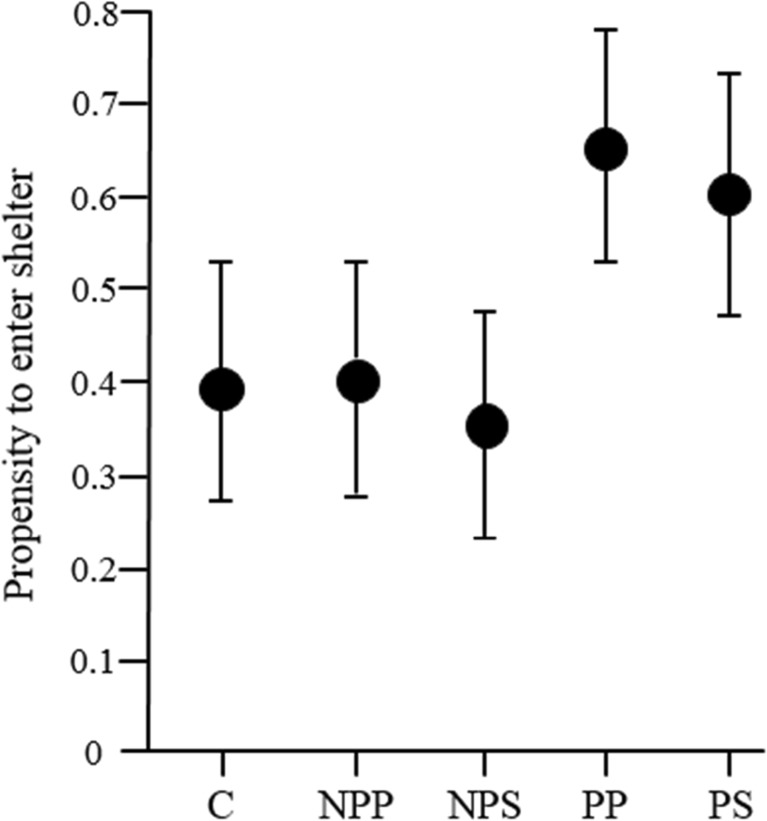
The propensity of test fish to enter the shelter (mean ± SE) after the flight response in the five treatments; higher values indicate a higher propensity to enter the shelter. C = control, NPP = herbivore picture, NPS = herbivore smell, PP = predator picture, PS = predator smell

None of the components of the escape response (response latency, burst-swim duration, hiding) or recovery from escape (time inactive, time in shelter) differed between the control treatment and the mean of all fish cue treatments [= contrast of C vs. (NPP, NPS, PP, PS); Tables 1, 2, 3, 4 and 5]. The comparison of the two predator cues, with the two non-predator cues [=contrast of (NPP, NPS) vs. (PP, PS)], revealed that fish exposed to predators had a shorter latency to respond towards the marble (Table 1, Fig. 2), a shorter burst-swim duration (Table 2, Fig. 3) and a higher propensity to enter the shelter after the escape (Table 3, Fig. 4), whereas the inactive time outside the shelter only tended to be shorter when non-predator cues were presented (Table 4) and the time spent inside the shelter after the escape response was not influenced by the treatments (Table 5). Interestingly, the sensory modality did not influence any of the measured parameters (= contrast of NPP vs. NPS and of PP vs. PS; Tables 1, 2, 3, 4 and 5). Only burst-swim durations tended to be shorter after fish were exposed to the herbivore image compared to herbivore smell (NPP vs. NPS; Table 2). The size of the test fish did not influence any component of the escape response or recovery phase (Tables 1, 2, 3, 4 and 5). The sex of the test fish only influenced the recovery time outside the shelter with males recovering faster than females (Table 4).

**Table 1:** Comparison of the latency to initiate a flight response

Factors	Estimate ± SE	*t* value	*p* value
Intercept	− 0.09 ± 0.414	− 0.23	0.822
C ➔ (NPP, NPS, PP, PS)	− 0.002 ± 0.004	− 0.59	0.556
(NPP, NPS) ➔ (PP, PS)	− 0.03 ± 0.008	− 3.71	< **0.001**
NPP ➔ NPS	− 0.007 ± 0.011	− 0.63	0.529
PP ➔ PS	0.004 ± 0.011	0.38	0.708
Size	0.190 ± 0.198	0.96	0.339
Sex	− 0.003 ± 0.026	− 0.12	0.901

All raw values were negative. To achieve a normally distributed error structure, data were transformed to positive values by reversing their sign to the opposite and subsequently square root transformed. Note that this results in estimates having opposite signs as well. Intercept estimates represent the grand mean of all treatments. Orthogonal comparisons of the treatments are displayed. The arrows indicate the direction of comparison within the contrast. The estimate value always refers to the treatment left of the arrow. If treatments are combined in parentheses, mean values of these treatments are used in the comparisons. The reference category for factor sex is ‘females’; *N* = 20 test fish in 100 trials, *p*-values < 0.05 are highlighted in bold

*C* control, *NPP* herbivore picture, *NPS* herbivore smell, *PP* predator picture, *PS* predator smell

**Table 2:** Comparison of burst-swim duration between onset and end of the flight response

Factors	Estimate ± SE	*t* value	*p* value
Intercept	0.646 ± 0.26	2.49	**0.026**
C ➔ (NPP, NPS, PP, PS)	0.003 ± 0.006	0.4	0.692
(NPP, NPS) ➔ (PP, PS)	0.05 ± 0.015	3.26	**0.001**
NPP ➔ NPS	− 0.037 ± 0.02	− 1.9	*0.061*
PP ➔ PS	0.026 ± 0.02	1.34	0.185
Size	− 0.055 ± 0.06	− 0.93	0.368
Sex	0.043 ± 0.033	1.32	0.207
Latency to respond	− 0.061 ± 0.164	− 0.37	0.713

Intercept estimates represent the grand mean of all treatments. Orthogonal comparisons of the treatments are displayed. For explanation of factors and interpretation of estimate values, see Table 1. *N* = 20 test fish in 100 trials, *p* values < 0.05 are highlighted in bold, *p* values 0.05 < *p* < 0.1 are italicised

**Table 3:** Comparison of the propensity to enter a refuge after the flight response

Factors	Estimate ± SE	*z* value	*p* value
Intercept	− 1.514 ± 5.343	− 0.28	0.778
C ➔ (NPP, NPS, PP, PS)	− 0.103 ± 0.111	− 0.93	0.351
(NPP, NPS) ➔ (PP, PS)	− 0.651 ± 0.251	− 2.59	**0.01**
NPP ➔ NPS	0.136 ± 0.354	0.38	0.702
PP ➔ PS	0.134 ± 0.351	0.38	0.702
Size	0.401 ± 1.212	0.33	0.741
Sex	− 0.713 ± 0.674	− 1.06	0.291

Intercept estimates represent the grand mean of all treatments. Orthogonal comparisons of the treatments are displayed. For explanation of factors and interpretation of estimate values, see Table 1. *N* = 20 test fish in 100 trials, *p*-values < 0.05 are highlighted in bold

**Table 4:** Comparison of the time spent inactive, but outside of the shelter, after the flight response

Factors	Estimate ± SE	*t* value	*p* value
Intercept	2.950 ± 3.723	0.79	0.445
C ➔ (NPP, NPS, PP, PS)	0.024 ± 0.049	0.50	0.622
(NPP, NPS) ➔ (PP, PS)	− 0.220 ± 0.120	− 1.83	*0.076*
NPP ➔ NPS	0.067 ± 0.151	0.44	0.662
PP ➔ PS	0.215 ± 0.195	1.10	0.277
Size	− 1.164 ± 2.329	− 0.50	0.627
Sex	− 0.667 ± 0.263	− 2.54	**0.028**

To achieve a normally distributed error structure, data were log-transformed. Intercept estimates represent the grand mean of all treatments. Orthogonal comparisons of the treatments are displayed. For explanation of factors and interpretation of estimate values, see Table 1. *N* = 20 test fish in 52 trials, *p* values < 0.05 are highlighted in bold, *p* values 0.05 < *p* < 0.1 are italicised

**Table 5:** Comparison of the time test fish stayed inside the shelter after the flight response

Factors	Estimate ± SE	*t* value	*p* value
Intercept	− 4.003 ± 9.100	− 0.44	0.667
C ➔ (NPP, NPS, PP, PS)	0.053 ± 0.113	0.47	0.643
(NPP, NPS) ➔ (PP, PS)	0.223 ± 0.232	0.96	0.345
NPP ➔ NPS	0.238 ± 0.389	0.61	0.545
PP ➔ PS	− 0.038 ± 0.288	− 0.13	0.896
Size	4.400 ± 5.760	0.76	0.458
Sex	− 0.462 ± 0.635	− 0.73	0.479

To achieve a normally distributed error structure, data were log-transformed. Intercept estimates represent the grand mean of all treatments. Orthogonal comparisons of the treatments are displayed. For explanation of factors and interpretation of estimate values, see Table 1. *N* = 19 test fish in 48 trials

## Discussion

As expected, *N. pulcher* flexibly adjusted key aspects of their escape response to the level of perceived danger as represented by predator or herbivore cues in our experiment. Contrary to our prediction, however, the escape responses of *N. pulcher* were unaffected by the sensory modality by which the cues were perceived. Thus, our results suggest that cues perceived by different sensory modalities can transmit similar information about danger posed by heterospecifics.

The ability to precisely discriminate between dangerous and harmless heterospecifics should provide animals with a considerable fitness benefit (e.g. Hirsch and Bolles 1980; Owings et al. 2001; Kullberg and Lind 2002). However, in aquatic environments, evidence that species behave differently when confronted with predator or non-predator cues remains mixed (Marsh-Hunkin et al. 2013; Palacios et al. 2016). This suggests that discrimination abilities either are species specific or depend on environmental features such as abundance and encounter rates of predators. More studies are needed to investigate the environmental conditions under which the ability to discriminate between heterospecifics favours survival prospects.

The efficacy of different sensory modalities in discriminating between dangerous and harmless heterospecifics has rarely been compared. Previous evidence from aquatic environments suggests that olfaction is of prime importance particularly in habitats, which have a constantly low visibility (Gerlai 1993; Kiesecker et al. 1996; Mathis and Vincent 2000). In seasonally fluctuating environments, be it aquatic or terrestrial, individuals might benefit from the use of multiple sensory modalities for predator recognition as shown in birds and mammals (Caro 2005; Saunders et al. 2013). In Lake Tanganyika, visibility varies greatly with season (Plisnier et al. 1999; Plisnier 2002). To cope with the fluctuating visibility, selection in *N. pulcher* might have favoured individuals evolving equally strong visual and olfactory acuity in predator discrimination. To the best of our knowledge, this is the first study to show that multiple sensory modalities can be equally important for predator discrimination in an aquatic environment. We propose that the role of environmental variability should be considered, if we aim at a comprehensive understanding of the use of different sensory modalities by animals.

Once predators are detected, moving away from an approaching predator is the most commonly observed antipredator behaviour in animals. Factors influencing the success of a flight response are the timing to initiate the response as well as flight speed (Godin 1997). In line with other studies manipulating the perception of safety of prey animals (for a review, see Lima and Dill 1990), we found that the latency to initiate a flight response and the burst-swim duration decreased if fish were exposed to a higher perceived predation risk. In our experiment, the shorter burst-swim durations may result either from a faster escape speed or from shorter escape distances. As all escape responses covered almost the same distance, starting at the same position and ending either inside the shelter or at its entrance, we argue that the shorter burst-swim durations more likely reflect a faster escape speed.

Following the startle response, *N. pulcher* had a higher propensity to enter a shelter if faced with predator cues. Entering a safe refuge after a threat stimulus has been documented in several prey species (for a review, see Kats and Dill 1998), which underpins the generality and efficiency of this response. In contrast, recovery times after the startle response were almost unaffected by perceived predation risk (except a weak tendency to be shorter inactive if not entering a shelter). This may be explained by the hunting strategy of the predator used in our study. *L. elongatus* is an ambush predator, which uses a fast, single-strike ‘surprise’ hunting strategy (Taborsky 1984). If the strike is unsuccessful, the predator moves on to another territory. Therefore, an increased waiting time before resuming normal activity after a strike may not yield substantial benefits, but it bears costs in terms of lost opportunities to feed and to defend the territory against intruders. Thus, an interesting avenue of future research would be to test whether recovery times of prey from attacks are systematically related to the hunting strategies of predators (e.g. Martin et al. 2010; Belgrad and Griffen 2016).

Hiding times in the shelter after the startle response and latencies to feed before the marble was dropped differed between sexes. Males left the shelter and started to feed earlier than females. Several alternative mechanisms might be responsible for this result, including sex-specific differences in personality (e.g. Schurch and Heg 2010), metabolic differences and ensuing differences in hunger state between sexes, or the different sex-specific roles and behaviours of male and female helpers (Mitchell et al. 2009) and breeders (Taborsky and Limberger 1981; Heg and Taborsky 2010).

In previous experiments, *N. pulcher* were shown to differentiate between herbivores and predators when defending their territories against live stimulus fish (Zöttl et al. 2013) or when responding to the visual display of animated images of these fish (Fischer et al. 2014). In this study, we extended the latter finding to a different context (flight responses) and we furthermore showed that *N. pulcher* are also able to distinguish between the two types of stimuli based on smell. The highly developed visual and olfactory discrimination abilities may be innate as the test fish were reared in the laboratory without contact to *L. elongatus* or *O. ventralis* prior to the experiment. Many prey species (including fish) do not show an innate antipredator response (Ferrari et al. 2005; Brown et al. 2013). However, genetically inherited antipredator responses were experimentally demonstrated in predator-naive larvae of the Lake Tanganyika cichlid *Simochromis pleurospilus* (Stratmann and Taborsky 2014). If predation risk is unpredictable, an innate predator response is only beneficial if individuals are not able to obtain personal or social information about the threat level (Brown et al. 2013). *N. pulcher* is a highly social cichlid living in large groups of up to 25 individuals (Heg et al. 2005b), thus potentially providing animals with opportunities to socially learn antipredator responses from their conspecifics. However, while the latter might be true, *N. pulcher* is exposed to a diverse but predictable predation pressure across populations (Groenewoud et al. 2016), which should render an innate predator response beneficial.

In conclusion, *N. pulcher* adjusted their escape response to the perceived predation risk, as they showed an improved performance during their flight response when predation threat was high. *N. pulcher* used both olfactory and visual cues to discriminate predators from harmless heterospecifics, and, interestingly, both senses seem to be equally efficient in this task. Our results suggest that individuals exposed to environments with fluctuating visual and olfactory conditions may rely on multiple sensory inputs for predator discrimination. Further studies are needed to investigate how the perceptual conditions of the environment affect the relative importance of different sensory modalities in animals.

## Electronic supplementary material

ESM 1 (XLSX 16 kb)
